# Infection Status Drives Interspecies Mating Choices in Fruit Fly Females

**DOI:** 10.1371/journal.pbio.0040345

**Published:** 2006-10-10

**Authors:** Richard Robinson

Hybridization is a constant possibility for two closely related species. Geographic isolation prevents interbreeding in some cases, but when the range of the two overlap, other mechanisms must come into play if they are to remain genetically distinct. Behavioral isolation is one such mechanism. If members of each group preferentially mate with their own kind, the two species can remain distinct even while residing together. Over time, such isolating behaviors may become more pronounced, and the genes governing them more widespread, a phenomenon termed “reinforcement.”

In evolutionary theory, reinforcement has typically been thought to act symmetrically on the two species. In a new study, however, John Jaenike and colleagues show that bacterial infection of one Drosophila species, but not another, and the resulting differences in hybrid viability, may account for highly asymmetrical reinforcement occurring in the two.

Wolbachia is a bacterium that infects many insect species, where it lives within the cells of the host, especially the ova and testes, and is transmitted from infected females to their offspring. Wolbachia infects virtually all members of the fruit fly species Drosophila recens, but not members of the closely related D. subquinaria. When an infected male D. recens mates with an uninfected female D. subquinaria, most offspring die in a process called cytoplasmic incompatibility. In contrast, however, when an infected female D. recens mates with an uninfected male D. subquinaria, the offspring are viable, and the hybrid females are fertile (the males are sterile, a typical result from cross-species hybridization).

To explore the effect of this difference on reinforcement, the authors began by establishing that the two species do indeed overlap in part of their range (a condition called sympatry), in central Canada, while maintaining separate populations elsewhere (allopatry). In the laboratory, uninfected D. subquinaria females from the region of sympatry never mated with D. recens males, while those from the region of allopatry did. They found no such pattern for infected D. recens females; instead, females from both regions were likely to mate with uninfected D. subquinaria males when placed together. The same discrimination or its lack was seen whether the females were presented with only one type of male (no choice conditions), or with males from both species, as might occur in the wild.

These mate-choice experiments illuminated two important phenomena. First, the most discriminating D. subquinaria females were those from populations living side-by-side with infected D. recens males. This makes sense, the authors suggest, given that less-choosy females that engage in such matings would leave few offspring, since almost all die off. Indeed, as the authors discovered, sympatric D. subquinaria females appeared to be so averse to mating outside their group that they also avoided mating with D. subquinaria males that came from the allopatric region. In contrast, allopatric D. subquinaria females, which have not been subjected to the same selective pressure, are not as discriminating. Second, D. recens females did not avoid interspecific matings nearly as strongly, since they also are not under the same selective pressure. Thus, the reinforcement process—the increase in mate discrimination—is highly asymmetric between the two species.

Finally, the authors asked whether the behavioral differences between sympatric and allopatric D. subquinaria females correlated with larger-scale genetic differences between the groups. They found it did not, and that overall there is considerable gene flow between the populations. This indicates that the differences in mate choice are likely the result of natural selection acting within the region of sympatry, rather than simple genetic isolation of the two populations. Interestingly, the reproductive isolation of the two D. subquinaria populations has been driven not by factors intrinsic to them, but by infection of entirely different species. It is possible that this isolation will ultimately lead to speciation within D. subquinaria, although the current high degree of similarity and existence of gene flow may suggest otherwise.

## 

**Figure:**
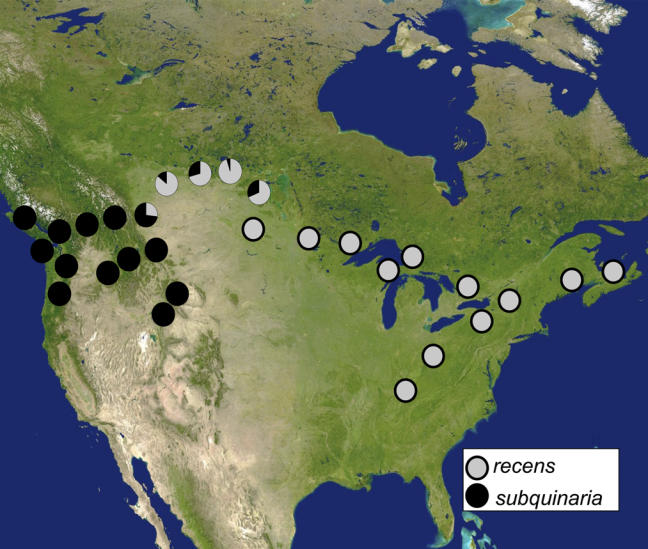
Geographical distributions of Drosophila subquinaria (black) and D. recens (gray), showing allopatric populations of D. subquinaria in the west and D. recens in the east, and sympatric populations in central Canada.

